# Acetic Acid-Catalyzed Formation of *N*-Phenylphthalimide from Phthalanilic Acid: A Computational Study of the Mechanism

**DOI:** 10.3390/ijms160612174

**Published:** 2015-05-28

**Authors:** Ohgi Takahashi, Ryota Kirikoshi, Noriyoshi Manabe

**Affiliations:** Faculty of Pharmaceutical Sciences, Tohoku Pharmaceutical University, 4-4-1 Komatsushima, Aoba-ku, Sendai 981-8558, Japan; E-Mails: kirikoshi@tohoku-pharm.ac.jp (R.K.); manabe@tohoku-pharm.ac.jp (N.M.)

**Keywords:** phthalanilic acid, intramolecular cyclization, *N*-phenylphthalimide, acetic acid catalysis, computational chemistry, double proton transfer, concerted bond reorganization

## Abstract

In glacial acetic acid, phthalanilic acid and its monosubstituents are known to be converted to the corresponding phthalimides in relatively good yields. In this study, we computationally investigated the experimentally proposed two-step (addition-elimination or cyclization-dehydration) mechanism at the second-order Møller-Plesset perturbation (MP2) level of theory for the unsubstituted phthalanilic acid, with an explicit acetic acid molecule included in the calculations. In the first step, a *gem*-diol tetrahedral intermediate is formed by the nucleophilic attack of the amide nitrogen. The second step is dehydration of the intermediate to give *N*-phenylphthalimide. In agreement with experimental findings, the second step has been shown to be rate-determining. Most importantly, both of the steps are catalyzed by an acetic acid molecule, which acts both as proton donor and acceptor. The present findings, along with those from our previous studies, suggest that acetic acid and other carboxylic acids (in their undissociated forms) can catalyze intramolecular nucleophilic attacks by amide nitrogens and breakdown of the resulting tetrahedral intermediates, acting simultaneously as proton donor and acceptor. In other words, double proton transfers involving a carboxylic acid molecule can be part of an extensive bond reorganization process from cyclic hydrogen-bonded complexes.

## 1. Introduction

**Scheme 1:**
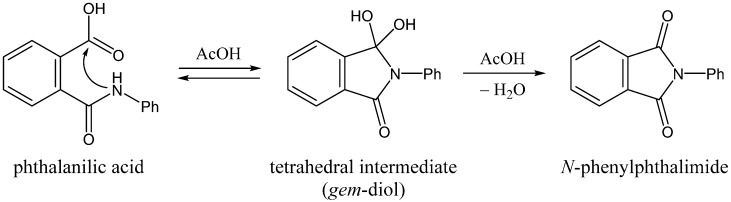
Two-step addition-elimination (cyclization-dehydration) mechanism for the formation of *N*-phenylphthalimide from phthalanilic acid in the presence of acetic acid (AcOH). Ph, phenyl.

Amide nitrogens are generally poor nucleophiles. This can be attributed to the amide resonance (*i.e.*, conjugation with the electron-withdrawing carbonyl group), which reduces the electron density of the nitrogen atom [1]. Indeed, examples of nucleophilic substitution reactions are rather rare in organic chemistry [2,3,4,5,6]. On the other hand, it has long been known that aspartic acid (Asp) and asparagine (Asn) residues in peptides and proteins tend to spontaneously cyclize to a succinimide species (both *in vivo* and *in vitro*), from which biologically uncommon β-Asp, d-Asp, and d-β-Asp residues can be formed [7,8,9,10,11]. These reactions are known to have relevance to aging and pathologies [9,10,11]. The succinimide formation from Asp and Asn residues is formally initiated by nucleophilic attack of the amide nitrogen of the *C*-terminal peptide bond of the Asp or Asn residue on the side-chain C_γ_ atom. However, it has not been fully understood why or how the peptide nitrogens can act as nucleophiles in the succinimide-forming reactions under relatively mild conditions [12,13,14,15]. This is why we have paid attention to the efficient formation of *N*-phenylphthalimide from phthalanilic acid in glacial acetic acid (Scheme 1) [2].

Perry and Parveen [2] showed that phthalanilic acid and its monosubstituents are converted to the corresponding phthalimides by heating with glacial acetic acid in relatively good yields. Their kinetics data support the two-step (addition-elimination or cyclization-dehydration) mechanism shown in Scheme 1. The first step, which was modeled as a rapid pre-equilibration, involves a reversible intramolecular nucleophilic attack by the amide nitrogen on the carboxyl carbon. This step was retarded by dilution of acetic acid with absolute ethanol, which indicates that the nucleophilic attack by the amide nitrogen is assisted by the solvent acetic acid. The observed intermediate, which was attributed to the *gem*-diol tetrahedral species, undergoes relatively slow dehydration in the second step to give the *N*-phenylphthalimide product. These results are very stimulating in relation to our recent computational findings that carboxylic acids (in their undissociated forms) can catalyze the succinimide formation from Asp and Asn residues [15,16].

In the present study, *ab initio* second-order Møller-Plesset perturbation (MP2) theory calculations were performed to show possible catalytic roles of acetic acid in the two-step formation of *N*-phenylphthalimide from phthalanilic acid. An acetic acid molecule was explicitly included in the calculations so that it can act as a proton mediator. Without such a proton mediator, the activation barriers would be prohibitively high corresponding to strained four-membered ring transition states. Our results show that the “nucleophilic attack” is only part of an extensive, concerted bond reorganization process occurring from a hydrogen-bonded complex between phthalanilic acid and acetic acid. The second step is also shown to be catalyzed by acetic acid.

## 2. Results and Discussion

**Figure 1:**
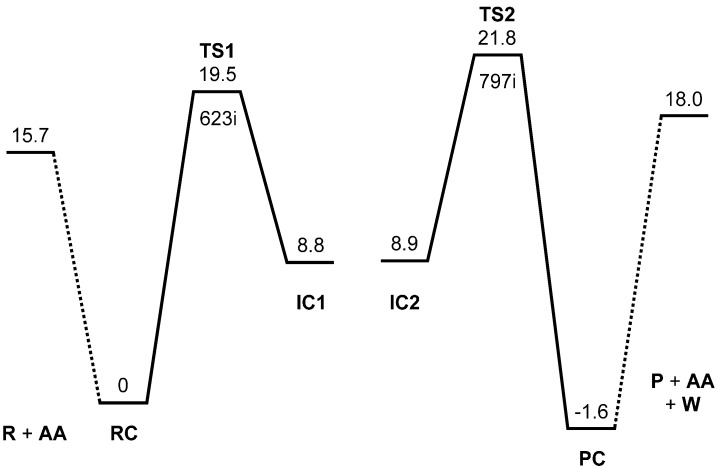
Energy diagram for the two-step formation of *N*-phthalimide from phthalanilic acid. MP2 (second-order Møller-Plesset perturbation theory) relative energies corrected for the zero-point vibrational energy (ZPE) are shown in kcal·mol^−1^ with respect to the reactant complex RC. R, reactant; AA, acetic acid; TS, transition state; IC, intermediate complex; P, product; PC, product complex; W, water. The imaginary frequency (cm^−1^) is also shown for TS1 and TS2.

**Figure 2:**
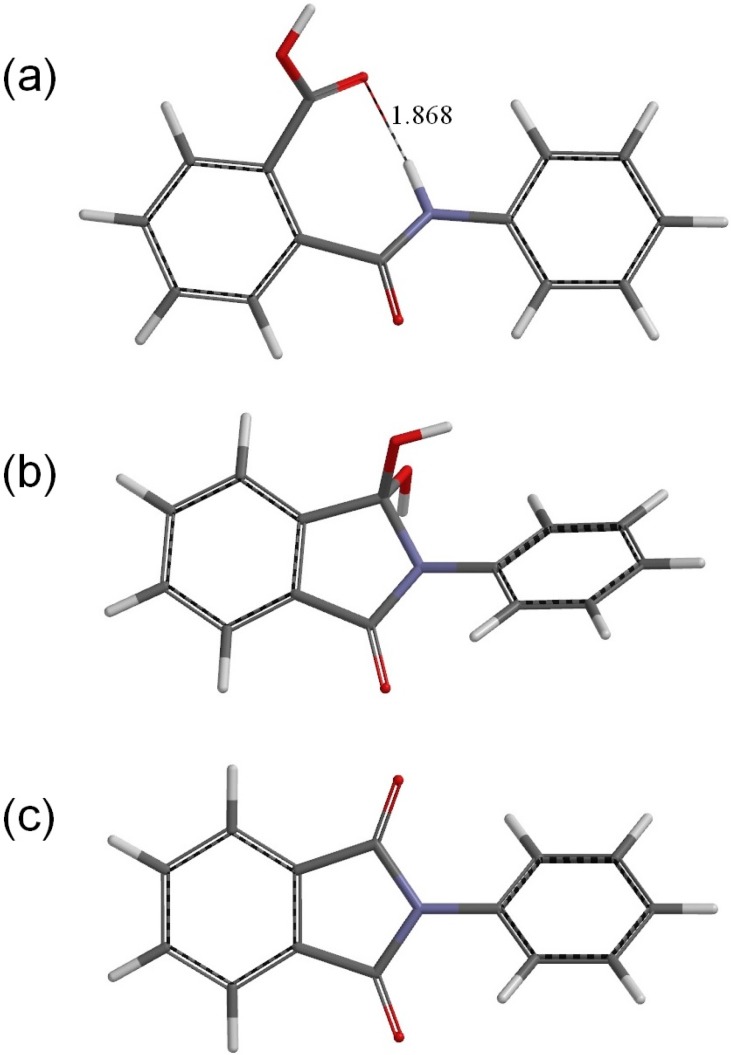
The geometries of (**a**) the reactant R (phthalanilic acid); (**b**) the *gem*-diol tetrahedral intermediate I; and (**c**) the *N*-phenylphthalimide product P. A hydrogen bond distance is shown in Å for R.

**Figure 3:**
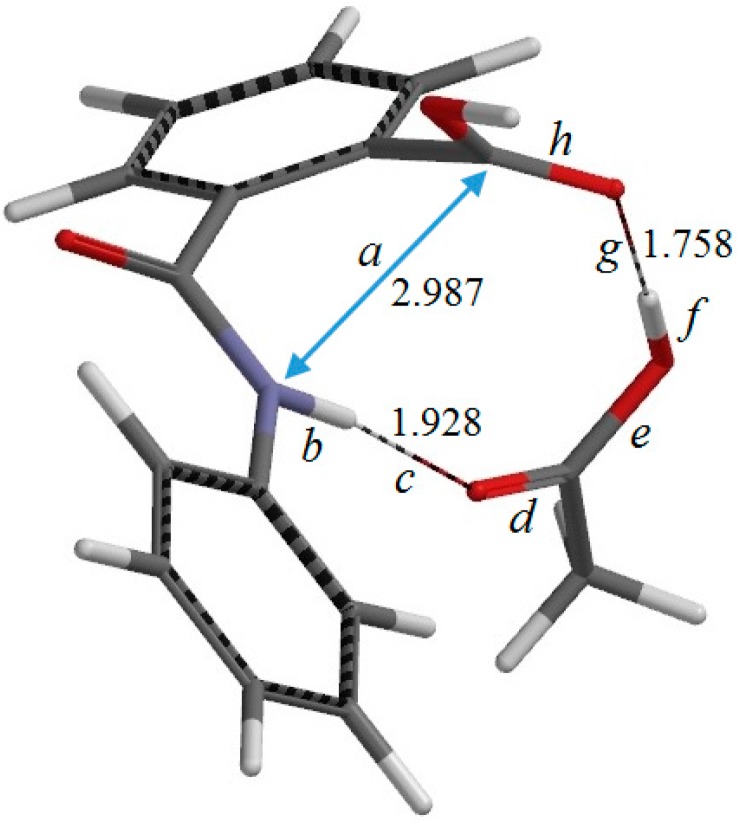
The geometry of the reactant complex RC (complex between phthalanilic acid and acetic acid). Selected interatomic distances are shown in Å. Also shown are the definitions of interatomic distances *a*–*h*.

**Figure 4:**
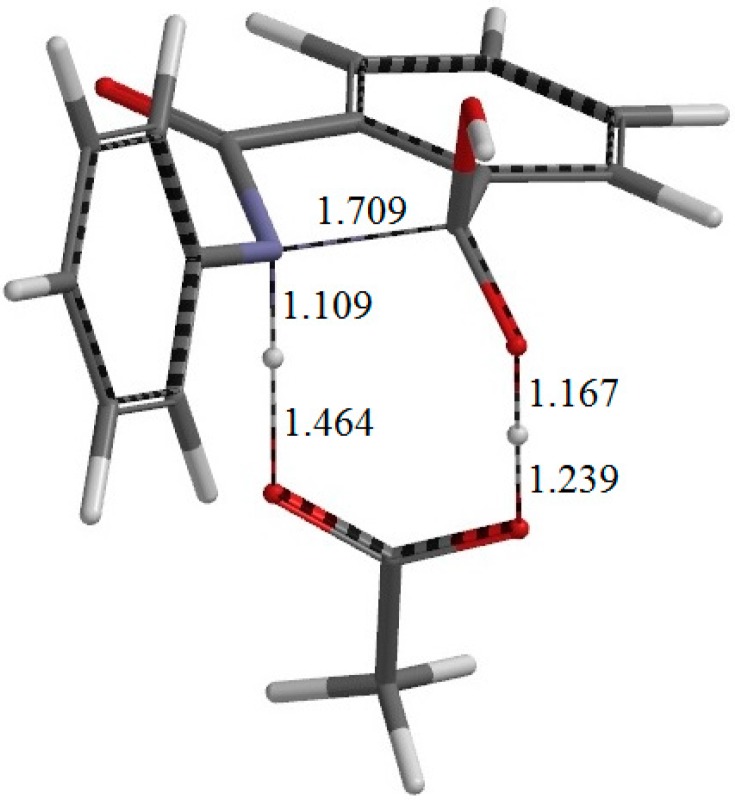
The geometry of the first-step transition state TS1. The distances of forming and breaking bonds are shown in Å.

**Figure 5:**
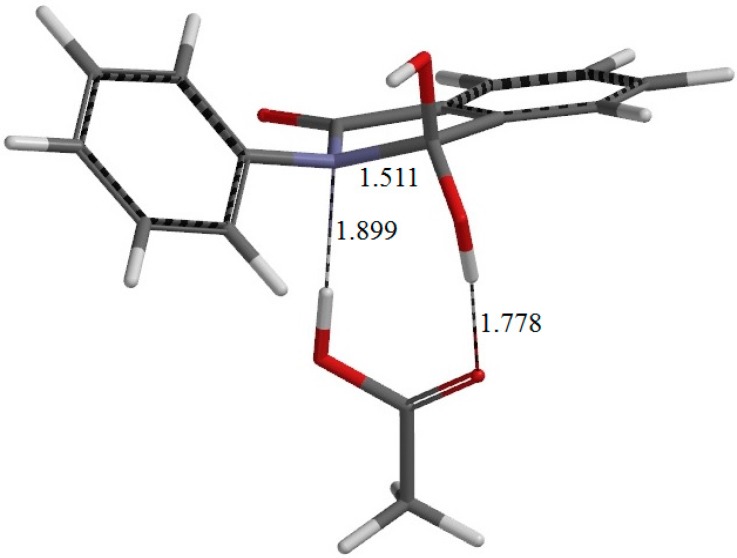
The geometry of IC1 (the intermediate complex directly connected to TS1). Selected interatomic distances are shown in Å.

**Figure 6:**
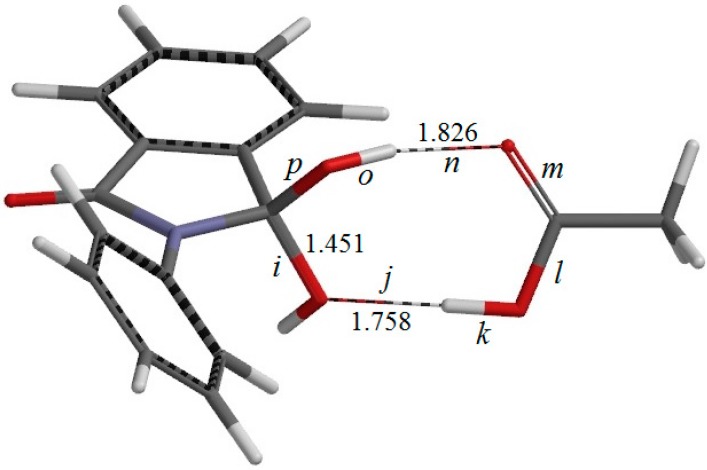
The geometry of IC2, the intermediate complex directly connected to TS2 (the second-step transition state shown in Figure 7). Selected interatomic distances are shown in Å. Also shown are the definitions of interatomic distances *i*–*p*.

**Figure 7:**
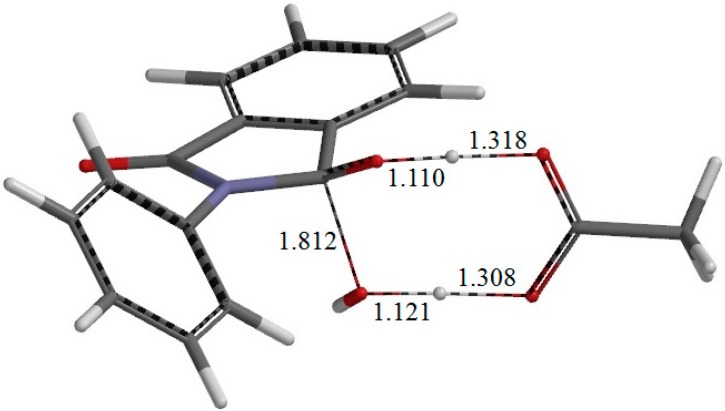
The geometry of the second-step transition state TS2. The distances of forming and breaking bonds are shown in Å.

**Figure 8:**
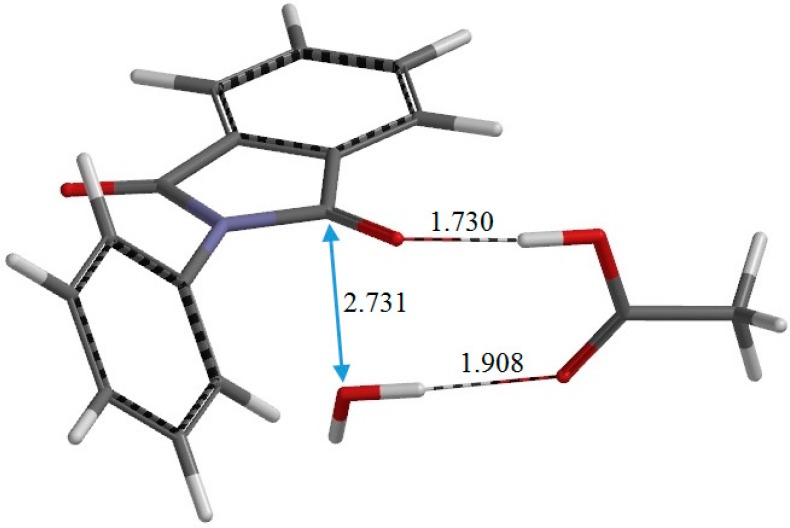
The geometry of the product complex PC (complex between *N*-phenylphthalimide, acetic acid, and water). Selected interatomic distances are shown in Å.

Figure 1 shows the MP2/6-31G(d,p) energy diagram for the acetic acid-catalyzed, two-step formation of *N*-phenylphthalimide from phthalanilic acid, and Figure 2, Figure 3, Figure 4, Figure 5, Figure 6, Figure 7 and Figure 8 show optimized geometries. All relative energies reported in the present work correspond to the electronic energy and are corrected for the zero-point vibrational energy (ZPE). Interatomic distances showing bond reorganizations in the first and second steps are tabulated in Table 1 and Table 2, respectively.

**Table 1:** Changes of the interatomic distances *a*–*h* (see Figure 3) (Å) in the first step.

Geometry	*a*	*b*	*c*	*d*	*e*	*f*	*g*	*h*
RC	2.987	1.017	1.928	1.228	1.337	0.988	1.758	1.231
TS1	1.709	1.109	1.464	1.267	1.283	1.239	1.167	1.309
IC1	1.511	1.899	0.988	1.337	1.230	1.778	0.982	1.387

**Table 2:** Changes of the interatomic distances *i*–*p* (see Figure 6) (Å) in the second step.

Geometry	*i*	*j*	*k*	*l*	*m*	*n*	*o*	*p*
IC2	1.451	1.758	0.988	1.334	1.233	1.826	0.980	1.374
TS2	1.812	1.121	1.308	1.273	1.277	1.318	1.110	1.291
PC	2.731	0.971	1.908	1.227	1.340	0.991	1.730	1.231

Figure 2a shows the optimized geometry of the reactant R (*i.e.*, the predicted most stable conformer of phthalanilic acid). This conformer is stabilized by an intramolecular hydrogen bond (1.868 Å) between the NH hydrogen and the C=O oxygen of the carboxyl group. Figure 3 shows the reactant complex (RC) formed between R and an acetic acid molecule AA. Upon the formation of RC, the intramolecular hydrogen bond in R is broken; instead, two intermolecular hydrogen bonds are formed between R and AA. One is between the NH hydrogen and the C=O oxygen of AA (1.928 Å), and the other is between the carboxyl C=O oxygen of R and the OH hydrogen of AA (1.758 Å). In RC, the amide and carboxyl groups of the phthalanilic acid moiety are twisted with respect to the 1,2-disubstituted benzene ring by about 51° and 44°, respectively; on the other hand, the phenyl group is almost coplanar with the amide moiety. The complexation energy between R and AA is 15.7 kcal·mol^−1^. This value, which is for the gas phase (and not corrected for the basis set superposition error), is not very important for the present discussion, considering that the actual reaction was conducted in glacial acetic acid [2].

From RC, cyclization occurs via the transition state TS1 (the transition state of the first step) (Figure 4) to give an intermediate complex IC1 (the intermediate complex directly connected to TS1) (Figure 5). In this step, a new single bond is formed between the nitrogen and carboxyl carbon atoms of phthalanilic acid to form a five-membered ring. The distances between these two atoms are 2.987, 1.709, and 1.511 Å in RC, TS1, and IC1, respectively. The forming N–C bond in TS1 is nearly perpendicular to the phenyl ring on the nitrogen atom.

The energy of TS1 relative to the reactant complex RC (*i.e.*, the intrinsic activation barrier of the first step) is 19.5 kcal·mol^−1^. For the 4′-methoxy-substituent of phthalanilic acid (where the primed number designates the *para* position of the phenyl ring), the corresponding rate constants were estimated at 317 and 333 K on the assumption that there is relatively small temperature dependence for the equilibrium constant of the reversible first step. From this, the Arrhenius activation energy can be calculated to be 21.6 kcal·mol^−1^. Considering the approximate nature of the experimental rate constants, the above activation barrier calculated for the unsubstituted phthalanilic acid seems to be reasonable.

**Figure 9:**
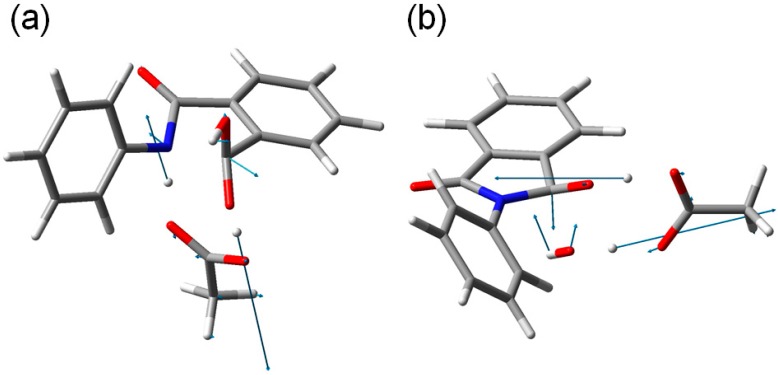
The transition vectors of (**a**) TS1 (the first-step transition state, Figure 4) and (**b**) TS2 (the second-step transition state, Figure 7).

**Figure 10:**
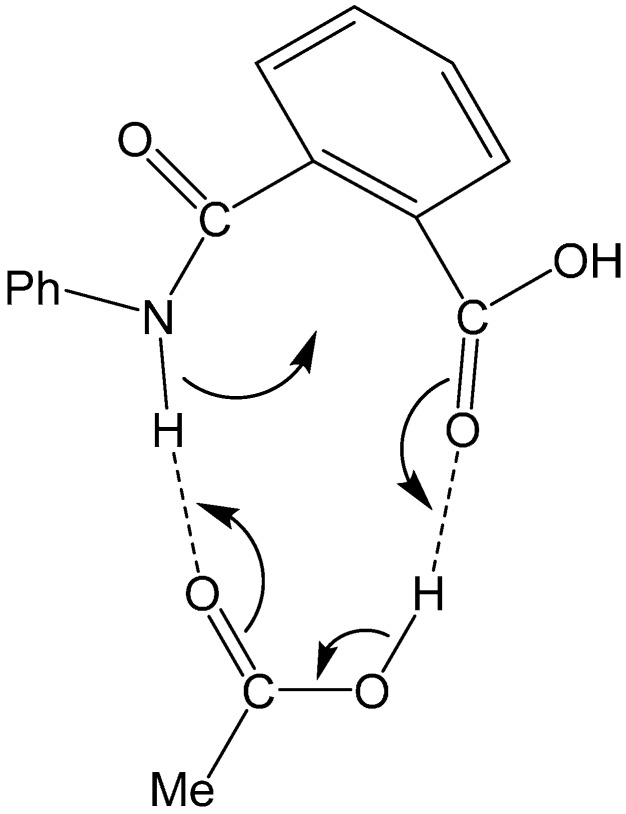
A curved-arrow representation of the extensive, concerted bond reorganization which occurs from the cyclic hydrogen-bonded reactant complex (RC) formed between phthalanilic acid and acetic acid. Dashed lines indicate hydrogen bonds. Ph, phenyl; Me, methyl.

Concomitantly with the N–C bond formation, a double proton transfer mediated by the AA molecule occurs, so that the resultant tetrahedral intermediate is a *gem*-diol species having two OH groups on the same carbon atom. This may be seen from the transition vector (*i.e.*, the vibrational mode corresponding to the imaginary frequency) of TS1 shown in Figure 9a and interatomic distances of RC, TS1, and IC1 shown in Table 1. More specifically, the NH hydrogen moves toward the C=O oxygen of AA, the OH hydrogen of AA moves toward the carboxyl C=O oxygen of phthalanilic acid, and the single and double bonds are interchanged in the COO moiety of AA. The AA molecule thus acts as both proton donor and acceptor in the double proton transfer. The bond reorganization in the first step can be represented as Figure 10. In the resultant intermediate complex IC1, the newly-formed AA molecule forms two hydrogen bonds to the intermediate molecule. One is between the amide nitrogen in the five-membered ring and the OH hydrogen of AA (1.899 Å); the other is between the C=O oxygen of AA and the hydrogen of the newly-formed OH on the five-membered ring (1.778 Å).

There exists another intermediate complex IC2 (the intermediate complex directly connected to the second-step transition state TS2) (Figure 6), which has almost the same energy as IC1. In IC2, the AA molecule now has two hydrogen bonds with the *gem*-diol group. More specifically, the C=O oxygen of AA forms a hydrogen bond (1.826 Å) to one of the OH groups in the *gem*-diol moiety, and the OH hydrogen of AA forms a hydrogen bond (1.758 Å) to the oxygen of the other OH group in the *gem*-diol moiety.

Figure 2b shows the optimized geometry for the isolated intermediate I. This was obtained from both IC1 and IC2 by removing the AA molecule and optimizing the remaining intermediate geometry. The gas-phase complexation energies for IC1 and IC2 are about 14 kcal·mol^−1^. Again, this value is not very important; better computational models with two or more AA molecules are expected to give a flat region on the potential energy surface corresponding to the intermediate.

From IC2, elimination of a water molecule occurs via TS2 (Figure 7) to give the product complex PC (Figure 8). The energy of TS2 relative to RC is 21.8 kcal·mol^−1^, which is higher than that of TS1 by 2.3 kcal·mol^−1^. This is consistent with the experimental finding that the second step is rate-determining [2]. In this step, one of the C–O bonds in the *gem*-diol moiety is cleaved. In TS2, the distance of the breaking C–O bond is elongated to 1.812 from 1.451 Å in IC2. Concomitantly with this bond cleavage, a double proton transfer mediated by AA occurs, as may be seen from the transition vector of TS2 shown in Figure 9b and interatomic distances of IC2, TS2, and PC shown in Table 2. The OH hydrogen of AA moves toward the departing oxygen, leading to formation of a water molecule. On the other hand, the hydrogen attached to the other oxygen of the *gem*-diol moiety moves toward the C=O oxygen of AA. In this double proton transfer, the AA molecule again acts as both proton donor and acceptor.

The resultant PC is a complex formed between the *N*-phenylphthalimide product P (Figure 2c), AA, and a water molecule W. In PC, the C=O oxygen and OH hydrogen of AA form hydrogen bonds to W (1.908 Å) and one of the carbonyl groups of the phthalimide moiety (1.730 Å), respectively. The complexation energy for the formation of PC from separated P, AA, and W is 19.6 kcal·mol^−1^. In IC1, I, IC2, TS2, PC, and P, the phenyl group is not coplanar with the fused ring. In P, which has *C*_2_ symmetry, the planes of the phthalimide and phenyl rings are twisted with respect to each other by 45°.

Both the first and second steps involve an extensive, concerted bond reorganization occurring from a cyclic hydrogen-bonded complex, as in the carboxylic acid-catalyzed mechanism of succinimide formation from Asp and Asn residues which we have recently proposed computationally [15,16]. For the first step, this bond reorganization may be represented as Figure 10. It seems that the size and shape of a carboxyl group are almost perfect to catalyze the intramolecular cyclization reactions to a five-membered ring by acting as both proton donor and acceptor simultaneously.

It should also be noted that both formation and breakdown of the *gem*-diol species occur in a single step due to the concertedness of the AA-mediated double proton transfers. Namely, any charged (protonated) form does not appear as a stable tetrahedral intermediate. This is consistent with the spectroscopic evidence that only one species accounts for practically all of the intermediate [2].

## 3. Computational Details

Energy-minimum and transition state geometries were located in a vacuum without any constraints by the MP2 method with the 6-31G(d,p) basis set. Vibrational frequency calculations were performed for all of the optimized geometries to confirm them as energy minima (with no imaginary frequency) or transition states (with a single imaginary frequency), and to obtain ZPEs. Intrinsic reaction coordinate (IRC) calculations were performed from the transition states followed by full geometry optimizations to confirm that each transition state connects two energy minima, as shown in Figure 1 by solid lines. All the calculations were carried out using Gaussian 09 [17], and optimized geometries were visualized by Spartan’14 [18]. The transition vectors of TS1 and TS2 (Figure 9) were displayed by using GaussView [19].

## 4. Conclusions

*Ab initio* MP2 calculations have been performed to show the detailed catalytic roles of acetic acid in the two-step formation of *N*-phenylphthalimide from phthalanilic acid. The calculated energetics were in reasonable agreement with experimental findings. The first step is intramolecular cyclization by bond formation between the amide nitrogen and carboxyl carbon atoms. Although this process is usually viewed as a nucleophilic attack by the amide nitrogen (which is a poor nucleophile), our calculation showed that the N–C bond formation is only part of an extensive, concerted bond reorganization involving a double proton transfer mediated by an acetic acid molecule. It should also be noted that only the neutral *gem*-diol form emerges as the tetrahedral intermediate. This is consistent with the spectroscopic evidence that only one species accounts for practically all of the intermediate [2]. The second step (dehydration of the *gem*-diol intermediate), which was calculated to be rate-determining in agreement with experiments, is also catalyzed by an acetic acid molecule. In both of the steps, the catalytic acetic acid molecule acts simultaneously as proton donor and acceptor.
